# Genetics of pubertal delay

**DOI:** 10.1111/cen.14606

**Published:** 2021-10-13

**Authors:** Tansit Saengkaew, Sasha R. Howard

**Affiliations:** ^1^ Centre for Endocrinology, William Harvey Research Institute, Barts and the London School of Medicine and Dentistry Queen Mary University of London London UK; ^2^ Endocrinology Unit, Department of Paediatrics, Faculty of Medicine Prince of Songkla University Songkhla Thailand

**Keywords:** delayed puberty, hypogonadotropic hypogonadism, puberty

## Abstract

The timing of pubertal development is strongly influenced by the genetic background, and clinical presentations of delayed puberty are often found within families with clear patterns of inheritance. The discovery of the underlying genetic regulators of such conditions, in recent years through next generation sequencing, has advanced the understanding of the pathogenesis of disorders of pubertal timing and the potential for genetic testing to assist diagnosis for patients with these conditions. This review covers the significant advances in the understanding of the biological mechanisms of delayed puberty that have occurred in the last two decades.

## 1 INTRODUCTION

**Figure 1:**
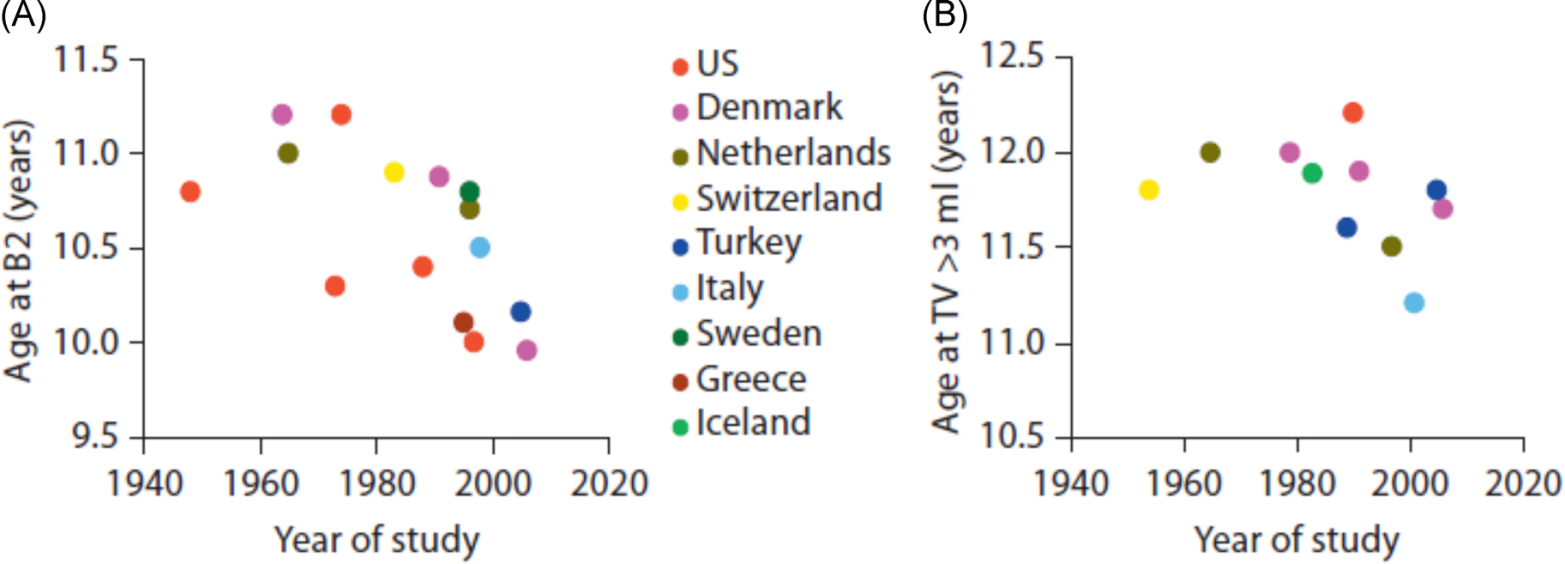
Secular trend in the mean age of onset of thelarche (A) and of testicular volume >3 ml (B). From Sorensen et al.^2^ [Color figure can be viewed at wileyonlinelibrary.com]

Delayed puberty (DP) is a common problem within the paediatric endocrinology clinic, affecting over 2% of adolescents. It is broadly defined as puberty commencing more than two standard deviations later than the mean age for the population.^1^ Due to the trend towards a decreasing age of puberty onset and the diversity in pubertal timing between populations (Figure 1), some experts have argued for adopting age cut‐offs for particular ethnic groups.^1^ Despite this, consensus remains that absence of breast development (Tanner stage B1) by the age of 13 years in girls and testicular volume remaining less than 4 ml (Tanner stage G1) in boys by the age of 14 years is consistent with a diagnosis of DP.^3^ Those patients with faltering progression through puberty, as identified by the use of puberty normograms, also need to be reviewed for conditions associated with DP.^4^

DP can be caused by a variety of aetiologies, including self‐limited DP (also known as constitutional delay in growth and puberty, CDGP, when associated with short stature for parental height), hypogonadotropic hypogonadism caused by a permanent or functional gonadotropin‐releasing hormone (GnRH) deficit, and hypergonadotropic hypogonadism due to gonadal insufficiency.^3^, ^5^ Self‐limited DP is the most common cause of DP, accounting for 63%–82% of boys and 30%–56% of girls presenting with DP.^4^, ^5^, ^6^ This condition is associated with a normal progression through puberty but at a timing later than the healthy population. Although self‐limited DP was thought to be a benign pubertal variant, many studies have found that this condition is associated with negative outcomes for adult health.^7^ Self‐limited DP is often seen in multiple generations of the same family, and 50%–75% of patients with self‐limited DP have a positive family history, suggesting a strong genetic basis.^8^ The inheritance pattern is complex, but autosomal dominant inheritance is predominant (with or without complete penetrance) and sporadic cases are also reported.^8^, ^9^

## 2 GENETIC REGULATION OF PUBERTAL TIMING IN THE GENERAL POPULATION

Evidence from twin studies has demonstrated that the timing of puberty is strongly heritable, and that genetic regulation is an important element in determining when healthy individuals enter puberty.^10^, ^11^ While environmental factors such as nutrition, emotional well‐being and geographical location influence pubertal timing, estimates from epidemiological data are that 50%–80% of the variation in age of pubertal onset is under genetic regulation.^12^, ^13^, ^14^ More recently, results of progressively larger genome wide association studies (GWAS) of age of menarche in women suggest that a large number of different genetic signals play a role in the range of pubertal timing that is observed in the general population.^15^ The signals identified to date explain ∼7.4% of the population variance in age at menarche, corresponding to ∼25% of the estimated heritability, and many have concordant effects on the age at voice breaking, a corresponding milestone in males.

## 3 GENETICS OF CENTRAL DP

### 3.1 Clinical phenotypes of congenital hypogonadotropic hypogonadism (CHH) and self‐limited DP

Self‐limited DP and CHH can present with the same phenotype, that is, delay entering puberty; however, these two conditions are different in clinical course and requirement for treatment. CHH, or Kallmann syndrome (CHH with anosmia), are pathological conditions with failure to progress through puberty which usually need intensive hormonal therapy, whereas self‐limited DP is generally a more benign condition once puberty is established, either after a period of monitoring or a short treatment course of sex steroids.^16^ ‘Red flag’ signs, such as micropenis or cryptorchidism in males, or other associated signs, can be a clue to the diagnosis of CHH; however, the majority of DP patients do not have an associated red flag feature at presentation. Moreover, both conditions may present with the same hormonal profile of hypogonadism with low gonadotropin concentrations.^1^ While traditionally, CHH and self‐limited DP were considered as two separate conditions, it is now apparent that there is a wide spectrum of phenotypes seen in clinical practice, ranging from complete CHH with lack of pubertal development, to partial hypogonadism with an arrest of pubertal development, reversible HH in some patients post treatment,^17^, ^18^ to isolated DP. Thus, accurate diagnosis for an individual presenting with central DP in adolescence is frequently challenging.

### 3.2 Genetics of CHH

To date, it has been shown that over 50 genes affecting the hypothalamic‐pituitary‐gonadal (HPG) axis contribute to the pathogenesis of CHH.^19^ These include factors regulating GnRH development, migration and maturation (*ANOS1*,^20^ *HS6ST1*,^21^ *PROK2/PROKR2*,^22^ *SEMA3A*,^23^ *SEMA7A*,^24^ *SEMA3E*,^24^ *PLXNA1*,^25^ *CCDC141*,^26^ *FEZF1*,^27^ *CC/NTN1*,^28^ *AMH/AMHR2*,^29^ *NDNF*,^30^ *SOX10*,^31^ *TUBB3*,^32^ *GLCE*,^33^ *FGFR1*,^34^ *FGF17*,^35^ *FGF8*,^36^ *IL17RD*,^37^ *DUSP6*,^35^ *FLRT3*,^35^ *SPRY4*,^35^ *KLB*,^38^ *WDR11*,^39^ *NR0B1* ^40^ and *CHD7* ^41^), regulation of GnRH neuronal activity (TAC3/TACR3^42^ KISS1/KISS1R^43^), and GnRH downstream function (*GNRH1/GNRHR*,^44^, ^45^ *FSHB* ^46^ and *LHB* ^47^). Different inheritance patterns, including X‐linked, autosomal dominant and autosomal recessive have been found. Moreover, 2.5%–15% of CHH patients have been reported to carry multiple deleterious variants in an oligogenic inheritance pattern;^35^, ^48^ with increasing discoveries the significant complexity of the inheritance of CHH is becoming more apparent. There are also numerous syndromic conditions associated with hypogonadotropic hypogonadism (Table 1), including *IGSF1* deficiency, which results in a syndrome of X‐linked central hypothyroidism with DP and macro‐orchidism in male patients.^65^

**Table 1:** Syndromic associations with congenital hypogonadotropic hypogonadism (CHH) or Kallmann syndrome (KS); Adapted from Howard^49^

Gene	OMIM ID	CHH	KS	Syndrome/syndromic features
*FGFR1/FGF8*	136350/600483	x	x	Hartsfield^50^
*LEP/LEPR*	164160/601007	x		Severe obesity^51^, ^52^
*PCSK1*	162150	x		Obesity, ACTH deficiency, diabetes^53^
*DMXL2*	616113	x		Polyendocrinopathy Polyneuropathy syndrome^54^
*RNF216/OTUD4*	609948/611744/212840		x	Gordon Holmes^55^
*PNPLA6*	603197	x		Gordon Holmes, Oliver Mcfarnlane,^56^ Lawrence Moon^57^
*SOX10*	602229		x	Wardenburg^31^
*CHD7*	608892	x	x	CHARGE^58^
*POLR3A/POLR3B*	614258/614366	x		4H^59^
*NR0B1*	300473	x		Adrenal hypoplasia^60^
*REV3L/PLXND1*	157900	x	x	Moebius syndrome^61^
15q11.2	176270	x		Prader Willi^a^^,^ ^62^
BBS 1‐11 (multiple loci) 20p12, 16q21, 15q22.3‐23, 14q32.1	209900	x		Bardet‐Biedl syndrome^63^
*PHF6*	301900	x		Borjeson‐Forssman‐Lehmann syndrome^a^^,^ ^64^
*IGSF1*	300888	x		X‐linked syndrome of central hypothyroidism, macroorchidism, and delayed puberty

^a^ Hypogonadism in these conditions may be hypogonadotropic, hypergonadotropic or a combination of both aetiologies.

### 3.3 Insights from CHH into the genetic basis of self‐limited DP

**Figure 2:**
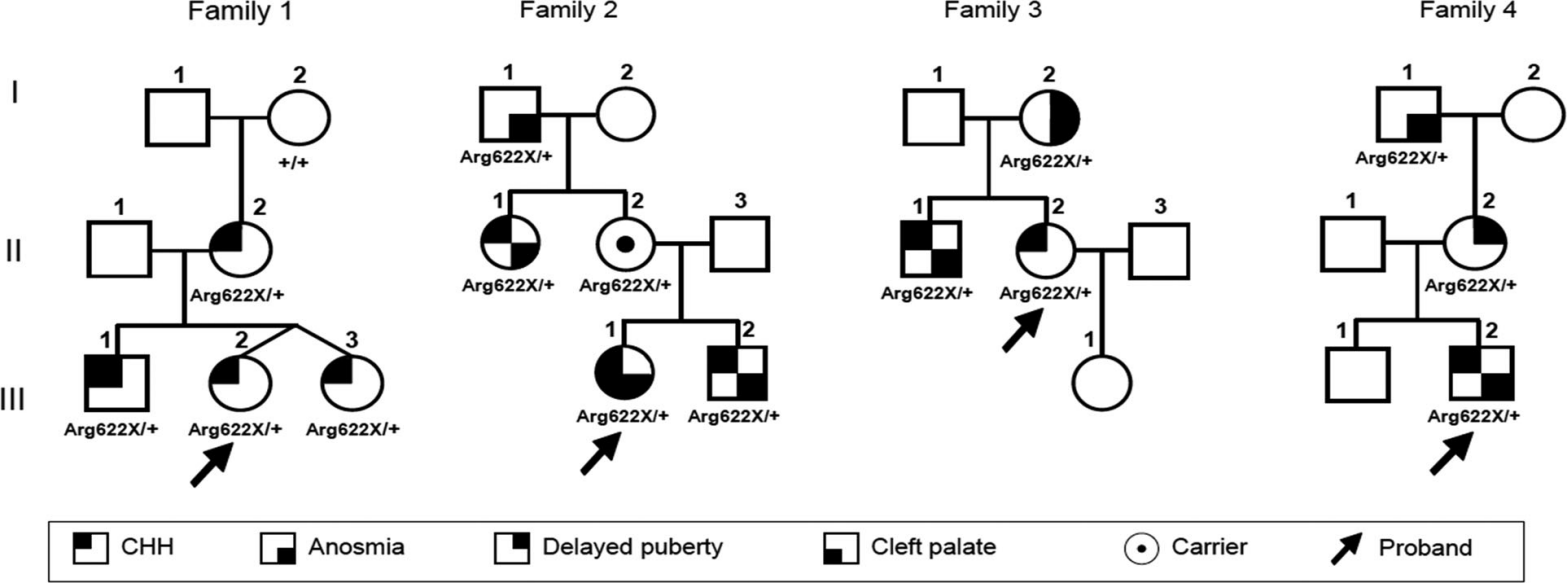
Example pedigrees of families with a loss‐of‐function mutation in *FGFR1*. In these families, individuals carrying the same mutation have a range of clinical phenotypes from Kallmann syndrome, to CHH and isolated DP. CHH, congenital hypogonadotropic hypogonadism. From Boehm et al.^66^ [Color figure can be viewed at wileyonlinelibrary.com]

**Figure 3:**
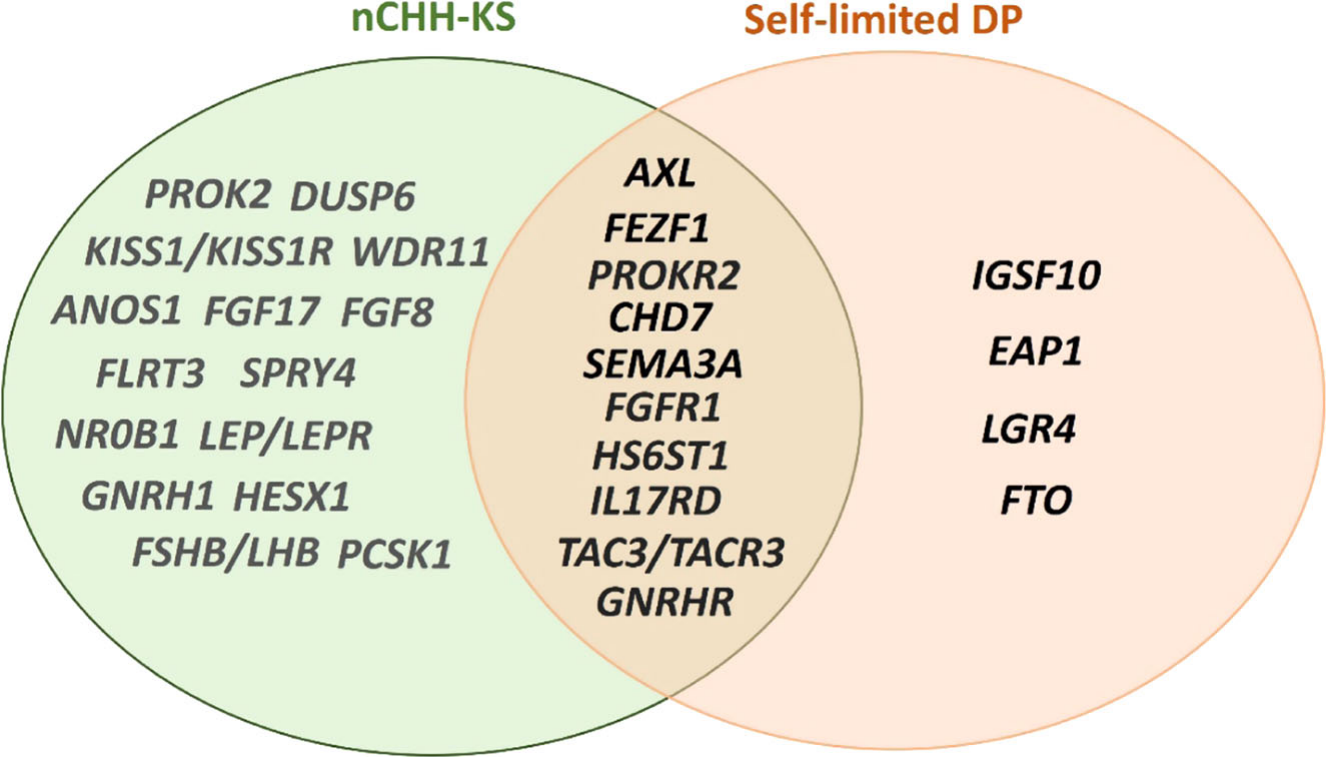
Overlap between genes identified in conditions with central DP. DP, delayed puberty; KS, Kallmann syndrome; nCHH, normosmic congenital hypogonadotropic hypogonadism [Color figure can be viewed at wileyonlinelibrary.com]

Although self‐limited DP is the most common cause of DP, the underlying genetic basis of this condition remains incompletely understood. The first information about the genetic inheritance of isolated DP was from patients with CHH or Kallmann syndrome (Figure 2), whose relatives were seen to have isolated DP, despite carrying the same genetic mutation as the proband with GnRH deficiency. Analysis of further CHH families suggested that self‐limited DP and CHH may share some overlap of their pathophysiology, with homozygous mutations in genes such as *GNRHR* ^67^, ^68^ and *TAC3* and its receptor^68^ causing CHH, while heterozygous carriage of the same variants was associated with the milder phenotype of self‐limited DP.^67^, ^68^, ^69^ Recently, a heterozygous mutation in a gene previously reported to cause CHH, *HS6ST1*, has been identified in a family segregating with pure self‐limited DP.^69^ In addition, analysis of a cohort of self‐limited DP (*n* = 72) identified rare and predicted deleterious variants in CHH genes including *AXL, FGFR1, HS6ST1, PROKR2, FEZF1* and *TAC3*, in patients with self‐limited DP^48^ (Figure 3). The mechanism by which these variants might contribute to a phenotype of isolated DP has not yet been fully elucidated, but may involve a reduction in the number of adult hypothalamic neurons or an impaired functionality of the GnRH neuroendocrine network, leading to a network that is less responsive to stimulation by upstream signals at pubertal onset with resultant delay.

### 3.4 New discoveries in self‐limited DP genetics

An increasing number of genes have been implicated in the pathogenesis of self‐limited DP over the last 5 years.^16^ Several strategies have been used to discover the genetic regulation underlying this condition, including interrogation of large cohorts of patients with isolated DP for potential mutations in genes relevant to the timing of puberty in the general population identified from GWAS studies,^70^ and for predicted deleterious variants in genes previously recognized from patients with CHH and Kallmann syndrome.^68^ The identification of the genetic basis of self‐limited DP has been accelerated by the use of next‐generation sequencing technology,^71^ although in a recent cohort review only 24% of cases with self‐limited DP who underwent whole‐exome sequencing had likely causal variants identified.^72^

**Figure 4:**
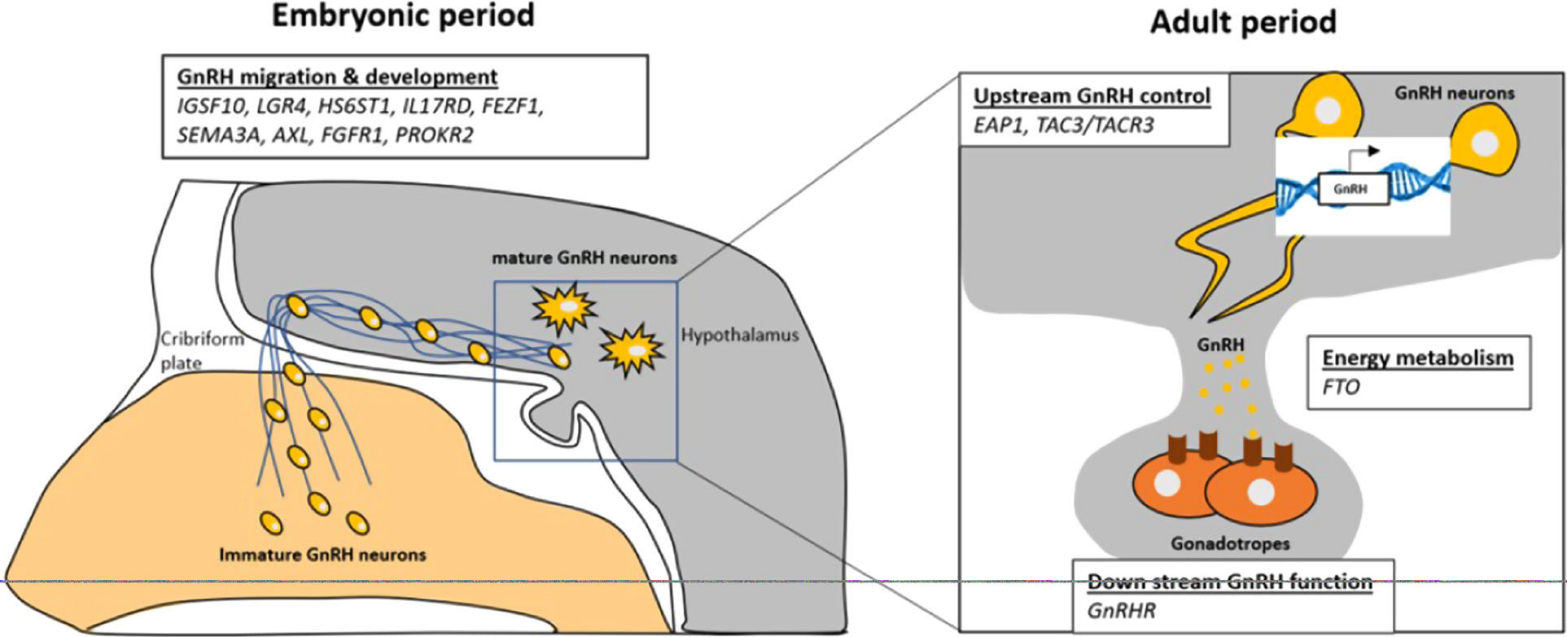
The genetic basis of self‐limited DP is related to GnRH neuronal development and function. DP, delayed puberty; GnRH, gonadotropin‐releasing hormone [Color figure can be viewed at wileyonlinelibrary.com]

To date, 14 genes have been identified as contributing to self‐limited DP, including those identified in relatives of CHH probands and others identified from large cohorts of familial self‐limited DP which have been extensively studied in vitro and in vivo. The majority of these genes have functions related to GnRH biology, including regulation of GnRH neuronal development and migration, GnRH upstream control, GnRH downstream action, and energy metabolism (Figure 4).

### 3.5 GnRH neuronal development and migration

GnRH neurons develop differently from other neurons in the hypothalamus, as they originate in the nasal placode but then migrate in the mid‐late foetal period towards the olfactory bulbs and across the cribriform plate to travel caudally into the basal forebrain (Figure 4, left panel).^73^ This neurodevelopmental process is controlled by a huge variety of cell matrix and adhesion factors, growth factors and others,^74^ the perturbation of many of which can result in a phenotype of CHH or Kallmann Syndrome.^75^

In some cases, defects in this process have been implicated in the pathogenesis of self‐limited DP. The first of these was the gene *IGSF10*, where two mutations in this gene were firstly identified in six families with self‐limited DP, and *Igsf10*‐knockdown in zebrafish embryos was shown to lead to impaired GnRH migration.^76^ After that initial discovery, mutations in*IGSF10* were identified in 11% of a familial self‐limited DP cohort.^48^ The postulated mechanism by which impairment of GnRH migration might result in self‐limited DP, is that this would impair the foetal development of the hypothalamic neuroendocrine network, due to a reduced number or delayed of arrival of hypothalamic GnRH neurons. In individuals with isolated DP one can hypothesize that this might result in a moderate impairment in function of the GnRH pulse generator, with a reduced but not absent ability to be reactivated during adolescence after the mid‐childhood dormancy, as is required for the onset of puberty.^76^

Following this, a heterozygous mutation in *HS6ST1*, a gene important for GnRH development via its interaction with ANOS1 and FGFR1,^21^ was found in individuals with self‐limited DP. Heterozygous deficiency of*Hs6st1*in a mouse model led to delayed vaginal opening with normal fertility in later life, similar to the phenotype of self‐limited DP patients. As this mouse model was found to have normal olfactory bulbs and a preserved number of GnRH neurons, the pathophysiological role of this gene in self‐limited DP was postulated to be via effects on GnRH neuronal activity or other downstream pathways of GnRH function.^21^, ^69^ Recently, mutations in the gene *LGR4* have been identified in families with self‐limited DP. In vitro studies demonstrated that the three *LGR4* mutations identified in patients with DP resulted in impairment of the Wnt‐signalling pathway, via effects on protein expression, trafficking, and degradation. The role of *LGR4* in GnRH development and migration was demonstrated by work in animal models, which showed that LGR4 deficiency led to abnormal GnRH migration in zebrafish embryos and delayed pubertal onset in mice.^77^ Once again, the absolute number of hypothalamic GnRH neurons was relatively preserved in mice with heterozygous knockdown of LGR4, suggesting that impairment of function not absolute number of GnRH neurons is responsible for the pubertal delay seen in human patients.

In addition, mutations in *IL17RD* were identified by direct sequencing in family members of CHH patients who have a phenotype of self‐limited DP. Previously, IL17RD has been shown to have a crucial role in GnRH neuronal migration via the FGF8/FGFR1 pathway. There have not yet been studies, in vitro or in vivo, to unpick the mechanism by which defects in this gene might lead to self‐limited DP.^68^

### 3.6 Upstream GnRH control

The activation of the HPG axis at the time of pubertal onset, after the long period of quiescence of this endocrine axis during mid‐childhood, requires optimal functioning of GnRH neurons in terms of GnRH transcription and secretion. This is modulated by several upstream transcriptional factors, including those with activating or repressing roles. At the onset of puberty, central inhibition of the GnRH neuroendocrine system decreases and there is a marked upregulation in the GnRH pulse generator activity. Kisspeptin signalling, one of the key stimulatory inputs to GnRH activity, is intensified at this time. In rodent models, increased kisspeptin synthesis in the KNDy neurons which directly synapse onto hypothalamic GnRH neurons, and an increase in the GnRH neuronal responsiveness to kisspeptin stimulation, has been well established,^78^ although this has not been verified in humans. Mutations in*KISS1* and its receptor *KISS1R* have been found to be responsible for disorders of pubertal timing including CHH.^79^, ^80^, ^81^ Moreover, a gain of function mutation in*KISS1R* was reported to cause central precocious puberty.^82^

Other important upstream regulators of GnRH transcription have been implicated in the timing of human puberty,^83^ but few have been demonstrated to be mutated in patients with DP. EAP1 is a nuclear transcription factor which trans‐activates the GnRH promoter and plays a part in regulating the timing of puberty. Mutations of *EAP1* were identified in self‐limited DP patients by whole‐exome sequencing (WES) analysis in a study which found that deleterious variants in this gene impaired its transcriptional activity on the GnRH promoter, resulting in reduced GnRH transcription and secretion.^84^ Furthermore, mutations in *TAC3* and *TACR3* have also been reported to cause self‐limited DP. These genes code for neurokinin B and its receptor, an important element of the KNDy neuronal complex which controls GnRH pulsatility.^85^ While heterozygous variants in *TAC3/TACR3* have been identified in self‐limited DP patients using WES,^68^, ^72^ they have not been tested in vitro or in vivo for pathogenicity.

### 3.7 Downstream pituitary action of GnRH

GnRH needs to bind to its receptor, GNRHR, to stimulate pituitary gonadotrophs to prompt gonadotrophin secretion. Abnormal GnRH‐GNRHR signalling has been demonstrated with *GNRHR* mutations which lead to the phenotype of CHH. Additionally, heterozygous mutations of *GNRHR* have been identified in patients who manifest only with self‐limited DP.^67^, ^72^ Interestingly, a partial loss‐of‐function mutation has been described in two brothers, one of whom had self‐limited DP followed by normal endocrine profiles and fertility in adult life, while the other required testosterone replacement on into adult life consistent with a diagnosis of CHH.^86^

### 3.8 Gene‐environment interaction

A wide range of environmental factors have been found to influence the timing of puberty. The effect of endocrine‐disrupting chemicals (EDCs) on pubertal timing have been widely studied. EDCs are environmental compounds that have a potential contribution to the observed shift towards an earlier onset of puberty in the developed world, Figure 1.^87^ Many EDCs are contained, and remain, within the food chain for many years, including pesticides [dichloro‐diphenyl‐trichloroethane (DDT), pyrethroids], polychlorinated biphenyls (PCBs), dioxins, and flame retardants [polybrominated diphenyl ethers (PBDEs)].

Epigenetic mechanisms have been implicated in the regulation of the timing of puberty. Experimental data from rats and goats give evidence for changes in DNA methylation and histone acetylation leading to altered gene expression during puberty.^88^, ^89^ In addition, there is evidence from mice models of the role of microRNAs (particularly the miR‐200/429 family and miR‐155) in the epigenetic upregulation of GnRH transcription during the infantile period of HPG axis activation (‘mini‐puberty’).^90^ Moreover, miR‐7a2, has been found to be important for HPG axis development. Deletion of mir‐7a2 causes hypogonadotropic hypogonadism and infertility in mice.^91^ Such epigenetic regulators are potential mediators of the effects of the environment on the hypothalamic regulation of puberty. However, the link between environmental factors and epigenetic control of puberty via the hypothalamus has not been fully clarified. Another epigenetic device, imprinting, has been identified to have a role in pubertal timing, with paternally inherited deleterious variants in *MKRN3* and *DLK1* identified in pedigrees with central precocious puberty.^92^, ^93^

### 3.9 Energy metabolism

*Fat mass and obesity‐associated protein* (*FTO*) had been implicated by GWAS to have role in the timing of puberty, and to impact on BMI and risk of obesity.^94^ Two rare deleterious variants in *FTO* were identified in 3 Finnish families from a self‐limited DP cohort.^70^ Patients who carried the variants had extremely low BMI since early life. Heterozygous *Fto*‐knockdown mice showed delayed pubertal onset. Although the mechanism by which *FTO* might influence pubertal timing is unclear, this may involve energy homoeostasis. FTO might act directly via the mTORC1 signalling pathway, which has role in energy balance and expression of kisspeptin in the hypothalamus, or it might affect BMI, thus influencing pubertal timing indirectly, or potentially exert an effect via both mechanisms.^70^ Leptin and its receptor, encoded by *LEP* and *LEPR* respectively, have an important role in mediating the relationship between energy metabolism and pubertal timing. Loss of function mutations in *LEP* or *LEPR* cause monogenic obesity.^51^, ^95^ These patients also display a CHH phenotype. Moreover, treatment with exogenous leptin can restore pubertal development in patients with loss‐of‐function variants of *LEP*.^96^

Murine models have provided further data linking metabolic inputs with pubertal timing. Overexpression of SIRT1 in a rodent model decreases Kiss1 mRNA expression and leads to DP.^97^ Alterations in nutritional status in this model led to changes in SIRT1 levels, mediating reorganization of the chromatin status and changes in histone methylation of the*Kiss1* promoter.^98^ Additionally, PACAP‐expressing neurons of the anterior hypothalamus have been proposed to play an important role in signalling nutritional state information to regulate GnRH release by modulating the activity of kisspeptin neurons, thereby regulating reproduction in female mice.^99^ However, there have not been reports of deleterious variants in these genes in patients with isolated DP.

## 4 GENETICS OF PRIMARY HYPOGONADISM

Patients with primary gonadal disorders may present with delayed or absent pubertal development. Turner syndrome is the most common form of hypergonadotropic hypogonadism in females, and puberty is usually absent, or otherwise delayed and followed by progressive ovarian insufficiency.^100^ About half of girls with Turner syndrome have a 45X karyotype, but mosaicism is also frequently seen. Other causes of primary ovarian insufficiency include: X isochromosome, where abnormal chromosome division results in duplication of identical chromosome arms, most commonly the long (q) arm; deletions and duplications of the short and long arm of the X chromosome; and mutations in genes including *BMP15, GDF9, FIGLA, FSHR, POLR3H, NOTCH2, FOXL2, AHM/R, FMR1, POF1B* and *DIAPH2*.^101^

In males, the commonest condition is Klinefelter syndrome (47,XXY), where individuals enter puberty spontaneously at a normal age, but testosterone levels become increasingly deficient by Tanner stages 4‐5.^102^ DP may be seen in those with a more complex karyotype (48,XXYY, 48,XXXY and 49,XXXXY). Several syndromes are associated with hypergonadotropic hypogonadism including Trisomy 21, hypogonadism associated with myopathies (myotonic dystrophy and progressive muscular dystrophy), Prader Willi,^103^ Werner^104^ and Alström^105^ syndromes.

Mutations in the gonadotropin receptors leading to hypergonadotropic hypogonadism are not a common cause of delayed or absent puberty. Loss‐of‐function mutations in the *LHCGR* gene present in females usually with primary amenorrhoea rather than DP.^106^ In contrast, males with *LHCGR* mutations lie along a phenotypic spectrum from disorders of sexual differentiation to undermasculinisation and infertility due to lack of testosterone secretion.^107^ Homozygous mutations in the *FSHR* are extremely rare, affecting mostly females with variable degree of pubertal development and complete ovarian failure. Point mutations in the extracellular domain of the *FSHR*, most frequently seen in the Finnish population, lead to subsequent inactivation of the receptor function resulting in raised FSH levels.^108^

## 5 CLINICAL UTILITY OF GENETIC DIAGNOSIS TO DISTINGUISH CONDITIONS OF DP

While a variety of clinical and biochemical investigations are available to assist with diagnosis of individuals with central DP, none of these can reliably distinguish CHH from self‐limited DP in adolescence.^109^, ^110^ Uncertainty in diagnosis has been reported to be associated with increased psychological stress for both adolescents and their parents.^111^ This is a vital clinical distinction to make, as if CHH is diagnosed, treatment modalities to allow optimisation of future fertility (particularly for boys) can be used—in the form of gonadotropins rather than sex steroids—for induction of puberty,^112^ and commenced earlier than the puberty induction regimen used for self‐limited DP patients. To date, more than 50 genes have been identified that carry mutations which lead or contribute to conditions of CHH.^16^ Similarly, as described, over the last 5 years an increasing number of genes have been discovered by next‐generation sequencing that underlie self‐limited DP. Crucially, while there is some overlap in the genetic background of these conditions, the majority of mutations are distinct between the two diseases.^48^ Therefore, genetic analysis can potentially be utilized to assist a clinician in distinguishing those adolescents with severe gonadotropin deficiency from those with isolated DP, allowing delivery of accurate and timely treatment to patients. Moreover, it can be helpful to facilitate appropriate counselling on likelihood of inheritance within families and for individuals undergoing fertility treatment. This is supported by a recent study of the use of targeted exome sequencing in the clinical setting to aid the differential diagnosis between CHH and self‐limited DP in a cohort of 46 adolescents presenting with severe pubertal delay.^72^

## 6 CONCLUSION

Accumulation of knowledge relating to the genetic basis of pubertal delay has greatly accelerated over the last two decades with the improvements in sequencing technologies. Identification of genetic defects underlying hypogonadotropic and hypergonadotropic hypogonadism have led to a greater understanding of the pathophysiology of these disorders. The genetic control of self‐limited DP is still mostly undiscovered, but from the initial findings it appears that the pathogenic mechanisms are related to GnRH neuronal development and biology, starting from neuronal development in the embryo to transcription and secretion of GnRH in the pubertal brain. The main pathophysiology of this condition is thus likely to be due to changes in responsiveness of the GnRH neuroendocrine system, resulting from defects of development of GnRH neurons and GnRH network functionality. Further developments will led to greater clarity on the biology of these conditions and the factors that determine timing of puberty in the healthy population, and can provide the opportunity for improved diagnostics and therapies for patients with disorders of puberty.
